# The Metaverse: A New Frontier in the Management of Hair Loss and Nail Disorders

**DOI:** 10.1111/jocd.16625

**Published:** 2024-10-08

**Authors:** Mohamad Goldust, Lidia Rudnicka

**Affiliations:** ^1^ Department of Dermatology Yale University School of Medicine New Haven Connecticut USA; ^2^ Department of Dermatology Medical University of Warsaw Warsaw Poland

Dear Editor,

The rise of the metaverse, a collective virtual space, has introduced new possibilities for healthcare, including dermatology [1]. Hair and nail disorders, which often carry significant psychological and social burdens, are areas where this technology may have transformative potential. However, concerns remain regarding its real‐world application and accessibility, especially for underserved populations, such as rural patients with limited access to advanced technology [2].

Although the metaverse holds promise for dermatology, particularly for hair and nail disorders, it is important to consider the current technological limitations. The promise of virtual consultations, enhanced by augmented reality (AR) and virtual reality (VR), offers patients new ways to engage with dermatologists remotely. In these consultations, patients could receive assessments for conditions such as alopecia or onychomycosis, potentially reducing the need for in‐person visits. However, although these technologies have the potential to offer high‐resolution, real‐time visualization, they are not yet ready for routine clinical use. For example, a virtual trichoscopy performed on a patient with androgenetic alopecia demonstrated promising early results in visualizing hair density, but ultimately still required follow‐up with in‐person imaging for confirmation. This highlights both the potential and the current limitations of virtual diagnostic tools. Current barriers include the precision required for dermatological assessments and the readiness of these technologies to provide accurate, reliable diagnostic information [3]. For instance, virtual trichoscopy or onychoscopy, while exciting, would require substantial advancements in imaging accuracy to replace in‐person evaluations.

A practical concern is the accessibility of such technologies to low‐profile patients, particularly in rural or underserved regions. The digital divide poses a significant obstacle to equitable access to metaverse‐based health care. High‐speed internet and advanced hardware, such as VR headsets, are often unavailable in these areas, leaving many patients unable to benefit from these innovations. A 2023 survey of rural healthcare access showed that fewer than 30% of patients had the necessary hardware for telemedicine, let alone advanced VR setups. Addressing this divide is crucial if the metaverse is to be widely adopted in dermatology. Government and healthcare organizations must work to improve digital infrastructure and provide affordable solutions that make these technologies accessible to all, regardless of geographical location or socioeconomic status [4].

Beyond accessibility, the metaverse's potential for improving clinical outcomes in hair and nail disorders must be carefully evaluated. Currently, the discussion surrounding its application in dermatology remains speculative, as there is a lack of empirical data demonstrating its effectiveness. The creation of AI‐powered avatars to simulate the progression of conditions like alopecia or onychomycosis is an interesting concept. Still, without real‐world examples or case studies demonstrating improved patient outcomes, this remains a theoretical advantage. Research must be conducted to evaluate the effectiveness of these virtual simulations in clinical decision‐making and their impact on treatment adherence and outcomes.

Virtual support communities within the metaverse offer another promising avenue for managing hair and nail disorders. Patients could access emotional support, share experiences, and consult with experts, potentially alleviating the psychological impact of these conditions. However, ensuring that these communities are inclusive and provide evidence‐based advice is essential. The anonymity and freedom of virtual environments can sometimes lead to misinformation, which could harm patient outcomes [5].

In conclusion, the metaverse holds the potential to transform the management of hair and nail disorders in dermatology, particularly through virtual consultations, AI‐driven simulations, and support communities. However, significant challenges remain, including technological precision, accessibility for underserved populations, and the need for empirical validation. A collaborative effort between healthcare providers and technology companies to conduct pilot programs in underserved regions could provide valuable data to overcome the digital divide and ensure the metaverse's benefits are universally available. For the metaverse to become a truly transformative tool in dermatology, these challenges must be addressed, and more robust, data‐driven evidence must be provided to support its use in routine clinical practice.

## Conflicts of Interest

The authors declare no conflicts of interest.

## Disclaimer

We confirm that the manuscript has been read and approved by all the authors, that the requirements for authorship as stated earlier in this document have been met and that each author believes that the manuscript represents honest work.

## Data Availability

Data sharing not applicable to this article as no datasets were generated or analysed during the current study.
